# The IMAGE project: methodological issues for the molecular genetic analysis of ADHD

**DOI:** 10.1186/1744-9081-2-27

**Published:** 2006-08-03

**Authors:** Jonna Kuntsi, Benjamin M Neale, Wai Chen, Stephen V Faraone, Philip Asherson

**Affiliations:** 1 MRC Social, Genetic and Developmental Psychiatry Centre, Institute of Psychiatry, King's College London, De Crespigny Park, London SE5 8AF, UK; 2 SUNY Upstate Medical University, 750 East Adams St., Syracuse, NY 13210, USA

## Abstract

The genetic mechanisms involved in attention deficit hyperactivity disorder (ADHD) are being studied with considerable success by several centres worldwide. These studies confirm prior hypotheses about the role of genetic variation within genes involved in the regulation of dopamine, norepinephrine and serotonin neurotransmission in susceptibility to ADHD. Despite the importance of these findings, uncertainties remain due to the very small effects sizes that are observed. We discuss possible reasons for why the true strength of the associations may have been underestimated in research to date, considering the effects of linkage disequilibrium, allelic heterogeneity, population differences and gene by environment interactions.

With the identification of genes associated with ADHD, the goal of ADHD genetics is now shifting from gene discovery towards gene functionality – the study of intermediate phenotypes ('endophenotypes'). We discuss methodological issues relating to quantitative genetic data from twin and family studies on candidate endophenotypes and how such data can inform attempts to link molecular genetic data to cognitive, affective and motivational processes in ADHD.

The International Multi-centre ADHD Gene (IMAGE) project exemplifies current collaborative research efforts on the genetics of ADHD. This European multi-site project is well placed to take advantage of the resources that are emerging following the sequencing of the human genome and the development of international resources for whole genome association analysis. As a result of IMAGE and other molecular genetic investigations of ADHD, we envisage a rapid increase in the number of identified genetic variants and the promise of identifying novel gene systems that we are not currently investigating, opening further doors in the study of gene functionality.

## Background

Research into the etiology of attention deficit hyperactivity disorder (ADHD) exemplifies the way that inter-disciplinary research fosters collaboration and opens up new avenues of investigation. International research has established that there is a strong genetically inherited contribution to ADHD and the genetic mechanisms involved are being sorted with considerable success by several centres worldwide. Recent review and meta-analyses of available data demonstrate an emerging set of findings that confirm prior hypotheses about the role of genetic variation within genes involved in the regulation of catecholamine neurotransmitters in susceptibility to ADHD [1,2]. Despite the importance of these findings, uncertainties remain due to the very small effect sizes that are observed, with average odds ratios in the range of 1.1 to 1.5. Under simple additive multi-gene models it is feasible that there exist numerous small genetic effects and we can estimate the contribution of the current loci to phenotypic variance (Table 1). Assuming an additive model, the variants identified so far explain around 3.3% of the variance, which is only 4% of the heritable component (assuming heritability for ADHD of 80%).

**Table 1:** Average odds ratios and 95% confidence (CI) from the pooled analysis of genetic variants found to be associated with ADHD in more than one study (Faraone et al., 2005) [1]. Quantitative trait effects are estimated for these key findings using the variance components 2 relative risk calculator . This program calculates the threshold, assuming a standard normal trait distribution, such that the QTL variance for the discrete category based upon this threshold would be the same as the QTL variance for the continuous measure. Assuming an additive genetic model, the proportion of phenotypic variance explained by the associated genes is around 3.2%. The number of families needed to replicate these findings with a nominal alpha of 0.05 and 80% is listed, in addition to the power from a sample of 200 families for the same significance level.

**Gene**	**OR**	**95% CI**		**Allele frequency**	**QTL**	**Number of families to replicate with 80% power**	**Power in sample of 200 cases and 200 controls**
DRD4	1.16	1.03	1.31	0.12	0.001	3196	0.115
DRD5	1.24	1.12	1.65	0.35	0.004	728	0.341
DAT1	1.13	1.03	1.24	0.73	0.001	2748	0.125
DBH	1.33	1.11	1.59	0.5	0.007	391	0.561
SNAP-25 (T1065G)	1.19	1.03	1.38	0.5	0.003	1043	0.253
SERT (HTTLPR)	1.31	1.09	1.59	0.6	0.006	466	0.490
HTR1B	1.44	1.14	1.83	0.71	0.010	315	0.652

However, it is possible that the observed effects do not reflect the true strength of the associations and we have merely detected one or more pointers, behind which lie larger genetic effects. Further work is required to establish the true size of the genetic effects and to use genetic information to refine the clinical and neurocognitive phenotypes associated with the genetic markers. Underestimates of effect size can arise for several reasons and a number of difficulties exist in identifying associated genes and deriving accurate estimates of effect size using genetic association studies. Some of the most likely causes are listed in Table 2 and discussed in more detail below. So until we have performed further investigations we cannot be confident that the genes identified so far do not make a more substantial contribution. In the following sections we will consider the effects of linkage disequilibrium, allelic heterogeneity, population differences and gene by environment interactions.

**Table 2:** Alternative explanations for small genetic effects in association studies of ADHD. This table lists potential explanations for small effect sizes in ADHD that range between 1.1 and 2.0. Studies to include or exclude each of these possibilities have yet to be completed, so the true size of the genetic effects remains unknown at this time.

Multiple genes of small effect	Main effect sizes of individual genes are small. Genetic influences consist mainly of common alleles, each making a small additive contribution to genetic effects.
Allelic heterogeneity	Average effect sizes of individual causal variants are small. The average effect size could be contributed by common variants, each conferring a small genetic effect and/or one or more rare variants conferring larger genetic effects.
Tagging markers (indirect association)	Strength of the observed association is proportional to the correlation between the genotyped marker(s) and the causal variant(s). This arises since not all the markers investigated are necessarily causal variants themselves, but may be tagging nearby functional genetic variants. The strength of the association will decrease with decreasing correlation between the tagging marker and functional variant.
Tagging phenotype	Strength of association is proportional to the correlation between the measured phenotype and underlying genetic liability. This arises since we do not know the best way to measure underlying genetic liability for a disorder. Phenotypic measurements are proxy variables that serve to tag the assumed underlying distribution of genetic risk. The strength of the association will decrease with decreasing correlation between the phenotypic measures and genetic liability
Interactions between adjacent loci	Variants within a gene may interact with each to alter gene function. This can arise since genetic variants may have functional consequences that depend on variation at a second variable site. An example that has been proposed is an interaction between the intro 8 and 3'UTR variants in the dopamine transporter gene (described in text).
Higher-order interactions	Main effects of individual genes may make little or no contribution to phenotypic variance. Genetic effects may be mediated by interaction with environment risks (gene by environment interactions) or other genetic loci (gene by gene interactions, referred to as epistasis).

### Linkage disequilibrium and direct versus indirect association

Across small intervals of the genome (10,000 – 100,000 base pairs), a phenomenon called linkage disequilibrium (LD) is observed. LD is the non-random assortment of alleles at two distinct loci, meaning that the genotype at a second locus can be marginally predicted by the genotype at the first locus. This non-random assortment gives rise to marginal information about a second locus from the genotype of a first locus. So, the genetic markers reported to be associated with ADHD may not be the causal variants (functionally significant variants or FSVs), but rather nearby genetic markers that are tagging true causal variants through LD. The strength of association between tagging markers (usually single nucleotide polymorphisms or SNPs) and the causal variant is directly proportional to r^2 ^(the squared correlation between two markers), a common measure of LD [3]. Further information about LD and its uses and measures are available [4-6].

Direct association is the analysis of the functional allele, whereas indirect association is the analysis of a secondary allele garnering marginal signal by means of LD with the functional allele. For example, for the genes listed in Table 1 there is evidence that genetic variants associated with ADHD in the dopamine D4 receptor gene (DRD4) and the serotonin transporter promotor region (HTTLPR) may alter the expression or function of the genes (reviewed in Asherson et al., 2004 [2]). In contrast, the genetic variants within or close to the dopamine D5 receptor (DRD5), synaptosomal associated protein (SNAP-25), dopamine beta-hydroxylase (DBH) and serotonin IB receptor (5HT1B) genes are not thought to alter gene function themselves. Rather, the variants that have been genotyped are in LD with, and therefore tag, nearby functional genetic changes that do alter protein structure or expression. Analysis of the dopamine transporter gene (DAT1) is ongoing and the functional status of the associated marker is not yet clear [7].

The extent to which direct or indirect association is the case for the variants hypothesized to be associated with a disorder such as ADHD, is difficult to assess. Biological significance cannot be declared as a result of a genetic association study but only through functional assays [8]. However, such assays are far more expensive to run and more difficult to interpret than genetic association. For this reason genetic association studies aim to cull the list of candidate variants to a likely few for biological investigation. Further information about likely candidates can be derived from haplotype analysis (the analysis of multiple closely linked markers). By typing multiple markers in a region we may be able to distill our list of suspects to a few variants on a specific haplotype.

Association with common genetic variants or haplotypes in ADHD has so far been interpreted to mean that a common causal variant exists that confers a small genetic risk for ADHD. Yet it remains feasible that rare alleles, which confer larger genetic risks in a subset of individuals, may exist if they are correlated with the common genetic markers or haplotypes that have been identified so far. With the exception of the dopamine D4 receptor gene [9-12], the genes thought to be associated with ADHD have not been fully investigated. For this reason we cannot say whether the genetic variants associated with ADHD are likely to be causal variants themselves or might tag common causal variants in the region, or even rare variants with a larger effect in a subset of individuals. The true strength of the association can only be estimated by direct association; this can only occur once we have a comprehensive understanding of how all variation affects each gene product.

**Figure 1:**
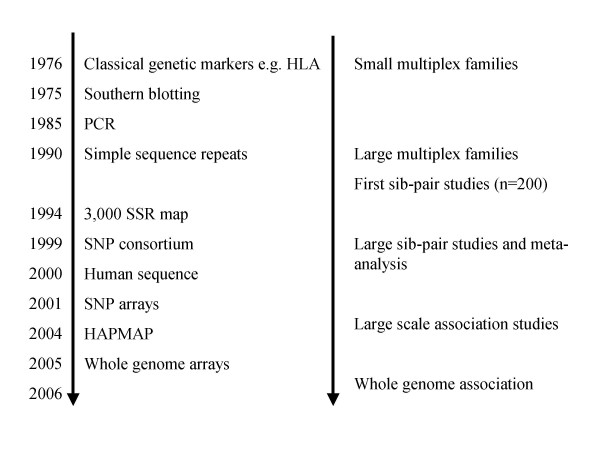
Historical perspective on gene mapping in common disorders. Initial studies, before DNA markers became available, relied on classical genetic markers such as blood or HLA types and therefore provided very limited information on a few regions of the human genome. The early genetic markers that used restriction enzymes to cut DNA at specific DNA sequences could identify sites that differed by one or more DNA bases. These restriction fragment length polymorphisms (RFLPs) were analyzed using a technique called Southern blotting that could identify one or a few markers at a time and was a relatively slow process. Linkage analysis came of age with the identification of another class of genetic variants, the simple sequence repeats (SSRs) that commonly consist of between two to four base pairs that are repeated in variable number tandem sequences (e.g. (AC)n) and are found approximately one every 50 thousand base pairs (Kb) across the genome. Around 3,000 such SSRs were identified for the first major human genome map in the mid 1990's, whereas only 400 of such markers are required for a first pass linkage scan. More recently the SNP consortium was established to identify single nucleotide polymorphisms (SNPs) that occur far more frequently, approximately one every 500 base pairs and are therefore useful for high-density association mapping. These are key to current studies since association, unlike linkage, can only be detected by markers that are correlated with functional variants in the population and are informative over very small distances. The HapMap project was set up to genotype SNPs across the genome in representative populations and establish the structure of linkage disequilibrium. High-density arrays that can be used to genotype between 350,000 – 500,000 SNPs in a single assay are now available and provide between 65–75% coverage for all SNPs with a minor allele frequency greater than 0.05. Further development of 1,000,000 plus arrays will be able to detect all common variation across the genome.

As polymorphism detection efforts increase and a higher density of genetic variants across genes become available, the chances of typing causal variants directly, or markers strongly correlated with such variants, is increasing (see Figure 1 for historical overview). Groundbreaking advances are being made in the rapid identification of SNPs throughout the genome [13,14] and efficient genotyping platforms that enable simultaneous genotyping of hundreds of thousands of SNPs are already available [15].

### Haplotype association in ADHD

A haplotype refers to a specific sequence along a single chromosome. For example, if we consider SNP markers, each with two possible alleles (A/a, B/b, C/c, D/d, E/e, F/f), there are 2^6 ^or 64 possible combinations along a single chromosome. However, for markers close together and in LD with each other (i.e. correlated with each other), there is usually limited haplotype diversity. This means that only certain combinations commonly occur. For example, if we assume equal allele frequencies of 0.5, the chances of the any single haplotype occurring (e.g. ABCDEF) would be 1/64. However, due to limited haplotype diversity we might find that 20% of chromosomes contain the ABCDEF haplotype and another 40% of chromosomes the AbCDef haplotype. Such markers are said to tag each within a population, since their relationship is non-random and they are associated with each other to form blocks or groups of highly correlated genetic changes.

**Figure 2:**
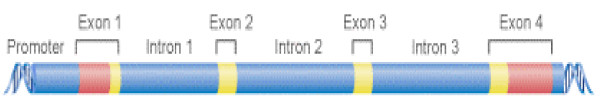
Illustration of a typical protein-coding gene. The promoter sequence regulates the process of messenger RNA (mRNA) production. mRNA is the template from which proteins are translated by matching of amino acids to the mRNA sequence. The gene is divided into exons (yellow), which are the coding regions for the amino acids in the protein. The untranslated regions (red) are found at either end of the mRNA and have various regulatory functions affecting mRNA expression and protein translation; because these regions appear in the mature mRNA molecule, they are also classified as exon sequences. Introns (blue) are found in the primary transcript and are spliced out to form the mature mRNA molecule. Sequences flanking each exon direct the splicing process. Additional elements regulating mRNA production can be found both within introns as well as outside of the gene. Genetic variation in any of the functional regions may alter either protein structure or expression.

**Figure 3:**
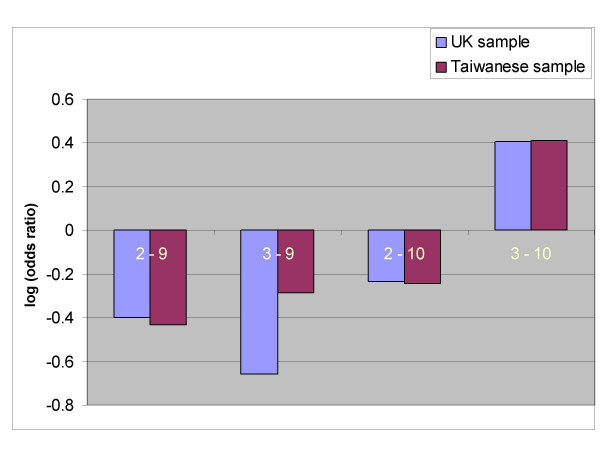
Log of the odds ratios for haplotype specific associations between ADHD and the intron 8 and 3'-UTR repeat polymorphisms in DAT1. Only chromosomes that contained the specific combination of the 3-repeat allele at the intron 8 marker and the 10-repeat at the 3'-UTR marker were over-transmitted from heterozygote parents to their affected offspring with ADHD (adapted from Brookes et al., 2005) [14].

As an example of a haplotype association in ADHD, we can consider recent findings from the analysis of the dopamine transporter gene (DAT1) and ADHD [7]. Several previous studies have documented the association of ADHD with a repeat length polymorphism in the 3'-untranslated region (3'-UTR) of DAT1, although averaged across studies the odds ratio is small and there is evidence of heterogeneity [2,16]. We recently genotyped an additional repeat length polymorphism within intron 8 of the same gene and found that in two independent populations from the United Kingdom and Taiwan, risk for ADHD was associated with a specific combination of alleles at the two loci. Only chromosomes with a 10-repeat allele in the 3-untranslated region and a 3-repeat allele in intron 8 were associated with risk for ADHD (see Figure 2 for explanation of these terms and Figure 3 for illustration of the haplotype specific association). Since none of the other common allelic combinations (10/2 and 9/3) conferred risk for ADHD, we concluded that either the 10/3 haplotype tags a functional variant that occurs on this haplotype background (i.e. neither of the markers studied so far are functional), or that there is a direct interaction between two functional variants. We subsequently reported a similar pattern of findings using the stage I sample of 680 families from the International Multi-centre ADHD Gene project [17].

The DAT1 haplotype association is illustrated in Figure 3. Although the most common explanation for these findings is the existence of a common variant of DAT1 conferring a small genetic risk to ADHD, it is also possible that a rare allele exists on the background of the 10/3 haplotype. For example, a rare risk allele (A+) conferring a large risk for ADHD might occur on chromosomes with the 10/3 haplotype. The 10/3/A+ haplotype would therefore be a relatively rare subset of all chromosomes with the 10/3 haplotype and would be associated with a large risk for ADHD, whereas chromosomes with the alternative 10/3/A- haplotype would confer no risk for ADHD. For these reasons we cannot be certain of the size of the genetic effect until the entire gene has been extensively investigated by re-sequencing and identification of all potential functional variants; either there is a small effect across the entire sample (consistent with multiple additive genetic effects) or a large effect in a subset of samples (consistent with genetic heterogeneity).

### Allelic heterogeneity and gene-wide tests of association

Allelic heterogeneity refers to a situation where multiple causal variants of differing frequency and effect size exist within a gene. Additionally, different populations may exacerbate this problem of allelic heterogeneity, as it is common for variation across the genome to be present or absent, depending on the population studied. Furthermore, LD, which is derived from population history, changes between populations [18]. The extent to which this is the case is still unclear, but a number of studies have observed different haplotype structures across different populations [19-21].

For these reasons, *gene-wide *testing that analyzes all sequence variation across a gene may provide a better frame for assessing evidence for association with a particular trait or disorder. Conceptually, there may be more than one variant within a gene conferring risk to a disorder such as ADHD. A gene-wide test of association takes this possibility into account by summing up all the evidence for association with markers occurring across an entire gene. At the same time gene-wide tests of association adjust significance values for multiple variants spanning a gene. *Gene-wide *testing therefore aims to allay some of the difficulties of allelic heterogeneity by allowing for multiple association signals to contribute to a single piece of evidence. Gene-wide tests of significance also provide a useful framework for dealing with the vast number of tests inherent in genome-wide association [22]. Fundamental to a gene-based approach is a comprehensive analysis of each gene, which requires re-sequencing in multiple individuals to identify all potential causal variants. Although at this time re-sequencing of all potential genes associated with ADHD is prohibitively expensive, it is envisaged that technical developments will make such an approach feasible within the next decade.

### Screening candidate genes for association

The candidate gene approach has been successful in identifying several genetic variants that are associated with ADHD. This has been largely a matter of good fortune, in the sense that the genes investigated initially were selected since they code for protein targets of many treatments used in general psychiatry, including stimulants used to treat ADHD. Such studies are, however, far from complete and in some cases candidate genes have been prematurely described as *not associated *with ADHD. This has occurred for two main reasons.

First, sample sizes used to date are insufficient to reliably detect small effect sizes, similar to those identified so far. Table 1 lists the sample sizes required to replicate the most significant findings reported to date, assuming 80% power of detection and a nominal alpha value of 0.05. We have also listed the amount of power at the same alpha level to detect these genetic effects with a sample size of 200, which is similar to that used in many published studies to date. Our most recent analyses of DAT1 and the dopamine D4 receptor gene (DRD4) in the IMAGE sample illustrate the problem. In a sample of 680 families we just hit nominal significance for the DAT1 association, whereas for DRD4 we required a total dataset of over 1,100 families [17]. For both associations the observed odds ratios were very close to those reported in the meta-analysis by Faraone et al. [1] (Table 1).

Second, few studies have taken a comprehensive approach to the analysis of individual genes by scanning genetic variation across entire gene regions. An example of a comprehensive gene-based approach is reported in a recent study of the noradrenergic transporter gene (NET1) [23]. This initial study aimed to screen the entire region spanning NET1. This was achieved by selecting database SNPs with minor allele frequencies greater than 5% that occurred within known functional regions; upstream promotor region, 5' and 3' untranslated regions, coding regions (exons) and intron sequences flanking each exon. The various sequences that make up the DNA sequence for typical protein coding genes are illustrated in Figure 2. Since NET1 has not been fully sequenced in multiple individuals, we do not know the location of all potential functional variants within the gene, which might for example include regulator elements in non-coding regions of the gene. Additional tagging SNPs were therefore selected, which were predicted to tag polymorphic variants (through LD) that are currently unknown and therefore not available for direct association analysis. In total we identified 26 SNPS and screened these for association with ADHD in case and control samples. Three SNPs were identified that showed nominal significance. Two of SNPs that had previously been reported to show no association with ADHD [24-26] were also negative in this study.

The small effect sizes that we observed for the three nominally associated SNPs led us to conclude that, after adjustment for the 26 SNPs tested, there was no evidence for association between ADHD and NET1. In a subsequent study, Bobb et al. [27] reported on genetic variants that had previously been reported to show nominal association with ADHD and found significant association with two of the three SNPs that we had identified (rs998424 and rs3785157), although with the opposite SNP alleles. The two markers associated with ADHD in both studies are strongly correlated with each other, having an r-square statistic of 0.93 in the UK sample and can therefore be considered to tag a single genetic association. However, despite evidence for association with the same two SNPs in two studies, we cannot be confident in these findings due the different directional effects of the SNP alleles. Further studies will therefore be required to clarify whether the SNP cluster tagged by these two markers is associated with ADHD or whether these are merely chance observations.

We have used a similar approach in the first set of analyses using the IMAGE stage I dataset of 680 families. Our aim in this study was to complete a systematic scan of genes that are functionally related to the main candidate gene systems identified to date. Our criteria for selection of candidate genes included analysis of genes with *a priori *evidence of association with ADHD and genes involved in the regulation of the neurotransmitter pathways implicated by the previous associations. We identified a total of 52 genes that fell into the categories of brain expressed catecholamine (dopamine, noradrenaline, serotonin) transporters, receptors, metabolism and catabolism genes. Additional categories included synaptic vesicle genes associated with synaptosomal associated protein gene (SNAP-25) and clock genes involved in regulation of circadian rhythms. In total we identified 1,536 SNPs with reported minor allele frequencies greater than 5% that fell within known functional sequences or tagged common haplotypes spanning each gene. Since some genes had a very high proportion of non-validated SNPs, we included 230 SNPs with non-validation status, of which only 13% turned out to be polymorphic. Of the 1,306 SNPs that were reported to be validated, only 68% were polymorphic in our sample, including 91% of 556 SNPs with genotypes available from Caucasian samples in the International HapMap database [13]; HapMap is an international resource for the selection of SNPs across the whole genome. Our final dataset included 928 polymorphic SNPs spanning 3,121 thousand bases pairs (kb) with an average SNP density of 1 every 3.36 kb. Despite the large sample size, we could only draw a few firm conclusions [17]. We found nominal significance with one or more genetic markers in eighteen genes, including the two most replicated findings in the literature: DRD4 and DAT1. Gene-wide tests adjusted for the number of markers studied in each gene identified associations with TPH2, ARRB2, SYP, DAT1, ADFRB2, HES1, MAOA and PNMT. Further studies will be needed to confirm or refute the observed associations.

### Gene by environment interactions

To date most genetic studies in ADHD have focused on the detection of genetic variants that have a main effect on the risk for behavioural disorders. However, it has been recognized for a long time that gene-environment interactions are likely to play an important role on risk for behaviour and in some cases will be present in the absence of main effects. What is not widely understood is that the heritable component estimated from family, twin and adoption studies indexes both the main effects of genes plus the effects of gene-environment interaction. For this reason environmental research remains critical to our understanding of psychiatric disorders, even for those that are highly heritable such as ADHD.

An emerging literature on the effects of gene-environment interactions on behavioural disorders and an outline of the methodological issues has recently been reviewed [28]. Using a longitudinal population sample from Dunedin in New Zealand, Caspi, Moffitt and colleagues reported three key findings. First they hypothesized that a functional polymorphism in the promoter region of the gene encoding the neurotransmitter metabolizing enzyme monoamine oxidase A (MAOA), would moderate the effect of child maltreatment in the cycle of violence. Their results showed that maltreated children with genotypes that conferred low levels of MAOA expression were more likely to develop conduct disorder, antisocial personality disorder and adult violent criminal behaviour than children possessing high activity variants of MAOA [29]. In the second study they hypothesized that a functional variant in the promoter region of the serotonin transporter gene (HTTLPR) would moderate the influence of stressful life events on depression. They found that individuals with 1 or 2 copies of the HTTLPR short allele exhibited more depressive symptoms, diagnosable depression, and suicidality following stressful life events than individuals homozygous for the long allele [30]. This finding has been replicated in several further studies and is now one of the most consistent findings in psychiatric genetics [31-33]. In the third study they reported that a functional variant of the catechol-*O*-methyltransferase gene (*COMT *Val158Met) would moderate the risk of cannabis use by adolescents on the later development of psychosis in adult life [34].

These three findings highlight the importance of considering the effects of environmental exposure in the search for genetic risk factors. Moffitt, Caspi and Rutter noted several important methodological points in their review [28]. First, they noted that several of their initial reports were subsequently replicated, indicating the robust nature of some G × E interactions on human behaviour. Second, that in each case the environmental risk involved had shown an association with the disorder in previous epidemiological studies. In other words, they were known environmental pathogens. Third, in several of the reports it was noted that there was no main effect of gene alone. This has important implications since the search for genetic associations with behavioural disorders would have been unsuccessful in these examples if interaction with the environmental pathogen had not been taken into account. These findings have promoted a new wave of interest in gene-environment research, although identifying such interactions remains a major challenge. Unlike the DNA variation, where we know that we will soon be able to scan the entire human genome for associated genetic variants, environmental research will depend on careful selection of appropriate and measurable environmental risks. Information ascertainment on environmental risk factors will present particular methodological challenge, as prospective cohort studies are likely to contain too few ADHD cases for meaningful analysis, while retrospective recall data on risk exposure in case and control design is likely to be confounded by recall bias. It is therefore particularly timely to examine the genetic contribution of ADHD quantitative traits amongst subjects from prospective cohorts, as well as gathering retrospective data of risk exposure in ADHD cases. Convergence of evidence from both prospective and retrospective data can then provide cross validation of findings from both strategies. Nevertheless, this will be necessarily a time consuming and costly process and requires reasonable prior hypotheses to be generated.

Once a specific gene-environment interaction has been identified, the next set of questions is to clarify the precise mechanisms involved. This is not always immediately obvious, since apparent interactions with an environmental variable may have several causes, including the possibility that scaling effects in the outcome measure can give rise to statistical interactions when the true mechanism is a simple additive effect [35].

As an example in ADHD research we consider the recent report of an interaction between the mothers use of alcohol during pregnancy and genetic variants of the dopamine transporter gene (DAT1) on the risk for development of childhood ADHD [7]. In this study only those individuals carrying the DAT1 risk alleles whose mothers used alcohol during the pregnancy showed an increased risk for ADHD. Yet there are several plausible explanations for this observation. First, there may be a direct toxic effect of alcohol on the developing fetus. Further work to establish this causal link needs to focus on more detailed analysis that considers the timing and amount of alcohol used by mothers during the pregnancy. However, other causal relationships need to be considered since maternal drinking may be correlated with parental behaviours that could act as more proximal risk factors, such as levels of critical comments, quality of parenting and maternal psychopathology including ADHD. Interactions with variables that reflect parental behaviour may also index genetic loading consistent with the increased co-transmission of interacting genes (gene-gene interaction also referred to as epistasis). For example, in this study we also found preliminary evidence for gene-environment correlation between the DAT1 risk alleles and prenatal use of alcohol. Although we controlled for this in our analysis, it highlights the complexity of interpreting gene-environment effects where genes cause change in parent as well as offspring behaviour. Well-designed epidemiological studies are one approach, but direct testing of environmental hypotheses may require the use of animal behavioural and genetic models and a focus on more direct neurobiological measures of brain function in addition to the analysis of behavioural phenotypes.

### 'Tagging phenotypes'

The term 'tagging phenotype' refers to the fact that the measures of ADHD used in genetic studies, such as DSM-IV diagnosis or symptom checklists, are unlikely to map directly onto the underlying distribution of genetic liability. The strength of the correlation between the measures used and genetic liability will therefore contribute to reduced estimates of genetic effect sizes. For this reason it has been argued that 'endophenotypes' (see below) that are thought to be more direct measures of brain processes than behavioural phenotypes might correlate more strongly with genetic risk factors for ADHD. Yet we cannot take this assumption for granted; evidence is as yet lacking. At this stage we advocate the use of cognitive endophenotypes to delineate the causal pathways that mediate genetic effects on behaviour, as discussed below, rather than their use as primary gene mapping tools.

### Cognitive endophenotypes

The term 'endophenotype' was adopted for use in psychiatric research by Gottesman and colleagues [36,37], who proposed the following criteria: (1) The endophenotype is associated with illness in the population; (2) The endophenotype is heritable; (3) The endophenotype is primarily state-independent (manifests in an individual whether an illness is active or not); (4) Within families, endophenotype and illness co-segregate, and (5) The endophenotype found in affected family members is found in non-affected family members at a higher rate than in the general population.

With the primary emphasis on delineating causal pathways, the study of cognitive endophenotypes represents a second stage in molecular genetic research on ADHD: once genetic associations are identified in the IMAGE and other ADHD samples, the *functionality *of the risk genes – the mechanisms by which the genes increase the risk for the disorder – becomes a key research focus. Such investigation of the cognitive and motivational processes in ADHD within a genetic design is at its early stages (for reviews of initial findings, (see [38-41]). For future progress, a careful selection of cognitive-experimental measures based on theory-driven phenotypic and quantitative genetic investigations is recommended.

Here, we discuss methodological issues relating to quantitative genetic data from twin and family studies on candidate endophenotypes and how such data can inform attempts to link molecular genetic data to cognitive, affective and motivational processes in ADHD. Processes that have been proposed to be affected in ADHD include state regulation, response inhibition, working memory, aspects of attention, temporal processing, 'delay aversion' and reward processing [42,43]; a consensus is yet to emerge on detailed aspects of the theoretical arguments.

### Estimating heritability and familiality

Gottesman and colleagues [36,37] included heritability as the second criterion in their list endophenotype criteria. As a clear distinction has not always been made between the terms 'heritability' and 'familiality', we first describe how such estimates are obtained.

A twin design is required to estimate heritability [44]. The logic behind quantitative genetic analyses of twin data has three parts. First, monozygotic (MZ) twins share all their inherited parental chromosomes and are therefore genetically identical, whereas dizygotic (DZ) twins, like ordinary full siblings, share on average only half of their parental chromosomes and therefore 50% of inherited genetic variation. For shared environmental influences MZ and DZ twins are expected to correlate to the same extent. As such, when the similarity of MZ twins is greater than the similarity of DZ twins, this indicates a genetic contribution to the behaviour being measured. In model fitting, this yields a 'narrow sense' heritability estimate (additive genetic variance). Second, if only genes were influencing their behaviour, MZ twins' behaviour should be at least twice as similar as DZ twins'. If, however, DZ twin pairs are less than twice as similar as MZ twin pairs, this indicates that environments the children share in common have enhanced their similarity. In model fitting, this yields an estimate for shared environmental variance. Third, if MZ twins, despite sharing all their genes, are not perfectly identical in their behaviour, this indicates that experiences unique to each twin have reduced the twins' behavioural similarity or the possibility of measurement error. In model fitting, this yields an estimate for child-specific environmental variance, which also includes measurement error.

The key difference between twin and sibling designs is that, whereas twin design produces the three estimates – heritability, shared environmental influences and child-specific environmental influences – sibling designs cannot distinguish between genetic influences (heritability) and shared environmental influences: 'familiality' reflects the combination of genetic and shared environmental effects.

### Heritability and familiality of candidate endophenotypes in ADHD

The cognitive-experimental variables that indicate significant heritability or familiality are targets for molecular genetic investigations. The existing twin and family data on measures of attention and executive functions were recently reviewed by Doyle et al [41].

A key methodological limitation in several of the previous twin studies relates to small sample sizes and therefore limited statistical power. Studies with somewhat larger sample sizes suggest a moderate degree of heritability for several measures of reaction time, working memory and general executive function performance [45-50]. We reported twin model fitting data at the Eunethydis 2005 meeting from 400 twin pairs on a go/no-go task, a reaction time task ('fast task'), digit span backwards and a 'delay aversion' task. Several key measures of reaction time, inhibition and working memory performance indicated a moderate degree of genetic influence [51]. These tasks are also being applied to a large sub-set of the IMAGE sample.

Yet we also demonstrated how the true extent of genetic influences may have been underestimated, due to possible effects on analyses from measure unreliability [51] (see also Luciano et al., [49]). This is because test-retest reliability sets an upper limit for the heritability estimates. Even a high test-retest reliability of .8 indicates that 20% of the variance cannot be accounted for; in twin model fitting the 20% would be 'added' to the variance component that reflects a combination of child-specific environmental influences and measurement error, hence deflating the heritability estimate (and possible shared environmental estimate). Given that many cognitive-experimental measures have at best moderate-to-good reliability (see, for example, Kuntsi et al., [52], caution is required when interpreting heritability estimates, especially across studies, if test-retest data are not available. A combination of test-retest reliability data and twin model fitting data can provide information on variables that will have maximum reliability and therefore maximum sensitivity to genetic individual differences [51].

### Multivariate quantitative genetic analyses

Beyond the initial requirement of heritability or familiality, an endophenotype for ADHD also needs to show *shared *genetic or familial influences with those on ADHD. This can be investigated using multivariate quantitative genetic analyses.

In multivariate twin analysis, MZ and DZ correlations are compared *across *traits: that is, one twin's scores on the first trait are correlated with the co-twin's scores on the second trait. If the cross-trait twin correlations are greater for MZ than for DZ twins, this implies shared genetic influences on the two traits. A genetic correlation (r_A_) indicates the extent to which genetic influences on one trait overlap with those on another trait (regardless of their individual heritabilities). Based on the genetic correlation and the individual heritability of each trait, the extent to which shared genetic influence generates the phenotypic correlation between the traits can be estimated.

In a sibling design, significant within-individual cross-trait correlations are indicative of common etiological influences on the traits; significant cross-sibling cross-trait correlations imply that these common etiological influences are familial. Where mean scores on a trait, such as a cognitive ability, are available from probands, their (unselected) siblings and controls, the extent of shared familial effects on the proband selection variable (such as ADHD) and the second trait (such as cognitive ability) can also be quantified using group familial correlation. This bivariate index, which varies between 0 and 1, indicates the extent to which the scores of the siblings on the second trait regress away from the population mean and towards the proband mean. Alternatively, if adopting a binary approach, the extent to which 'unaffected' siblings of probands show the second trait (such as poor performance on a cognitive task) can be studied as an indicator of an endophenotype.

Initial data suggest shared genetic or familial effects on ADHD and response variability and aspects of executive function performance. The finding on shared genetic/familial effects on ADHD and response variability was first suggested by our small-scale twin study [53] and has subsequently been replicated in an independent family study [54] as well as, most recently, in a sub-set of the IMAGE sample (unpublished data). Further possible shared familial effects with ADHD have been indicated for performance on tasks measuring inhibition and set shifting [55,54].

Multivariate quantitative genetic analyses can also be used to investigate the extent of shared genetic influences across multiple cognitive-experimental variables. Such analyses can be informative, both theoretically regarding possible causal pathways, and in guiding molecular genetic analyses. If meaningful composite scores can be created, these are likely to have increased reliability compared to the original scores. Such composite scores would also help reduce the need for multiple comparisons within a molecular genetic investigation.

### The IMAGE project

These are exciting times in the world of human molecular genetics. The sequencing of the human genome in 2000 has been rapidly followed by the development of international resources for whole genome association analysis. Micro-array technology is already available for the simultaneous analysis of 500,000 SNP markers across the human genome and these resources will be further developed in years to come.

The International Multi-centre Gene (IMAGE) project is well placed to take advantage of these emerging resources. The IMAGE sample consists of a European multi-site project with a final projected dataset of 1,400 families. Clinical data and cryopreserved lymphocytes or established cell lines are deposited at Rutgers University, to ensure the dataset will be available to international investigators now and in the future. The overall aim of the IMAGE project is to take a systematic approach to screening the genome for novel genes and gene systems using a combination of categorical and quantitative trait approaches to linkage and association. This dual approach is well supported by recent findings that suggest the existence of genes of moderate effect size (e.g. odds ratios > 3) co-acting with multiple genes of small effect (e.g. odds ratio < 3). A quantitative trait locus genome linkage scan is planned for summer 2006, which we propose to follow with genome association studies using the latest generation of high-density SNP arrays.

The sample consists of European Caucasian subjects recruited from twelve specialist clinics in eight countries: Belgium, Germany, Holland, Ireland, Israel, Spain, Switzerland and United Kingdom. The initial stage I sample consists of 680 DSM-IV combined type probands with 808 siblings, of which 102 also had combined type ADHD, making a total of 782 affected individuals. Since we evaluated all available siblings, which included 102 combined type probands from 808 siblings recruited into the study, we estimated the λ_s _value (risk to siblings/population prevalence) for combined subtype to be around 6%, using the population prevalence estimates from a recent survey in the United Kingdom [56]; a similar estimate to that reported in dizygotic twins by Todd et al. [57]. Although the DSM diagnostic criteria were not designed to be genetically homogeneous categories, the analysis from Todd and colleagues suggests that DSM-IV combined type ADHD may be a genetically homogeneous subgroup, since this subtype falls within a single empirically derived latent class that shows high levels of subtype concordance in monozygotic and dizygotic twin pairs.

Although the IMAGE sample is a relatively large clinical sample, we have seen that association signals have been very small. For this reason we should expect that at a genome-wide level, positive association signals will still not stand out above the background distribution of association findings. Larger scale collaborative projects will therefore be required if we are to find many of the genes, particularly those that fall within novel genes and gene systems, that influence risk for ADHD. We (and others) therefore aim to establish international collaborations to generate the very large datasets, in the order of two to five thousand samples, for whole genome analysis.

### Concluding remarks and future directions

As a result of these new studies, we envisage a rapid increase in the number of identified genetic variants associated with ADHD and the promise of identifying novel gene systems. The size of the genetic effects ADHD identified to date are small, although as we have discussed in some cases these may be tagging larger effects. Translation of these effect sizes into 'real world significance' is difficult to determine in advance of completing the task of gene identification. Clearly, if one or more genetic variants are identified with a large influence on risk for ADHD, these might play a key role in diagnostic prediction and forming targets for drug development.

However, it is entirely feasible that larger genetic effects will not be identified and the risks from any single genetic variant will be small and have little predictive value on their own. In this scenario diagnostic predictions would likely be expressed as a probability depending on the additive effects of multiple genetic variants. It is, however, expected that the additive effects of genes will compromise the function of only one or a few neurobiological pathways, and the identification of even small genetic effects will help to identify the key pathways involved, thereby identifying novel systems for drug discovery and investigation of critical interactions with environmental risks. Clearly the current data implicate genetic variation of dopamine pathways in the etiology of ADHD, although this finding was predicted in advance of the genetic studies and does not therefore come as a great conceptual advance in the field. The key additional question is whether genetic approaches that do not rely on a priori hypotheses will uncover novel genes and neurodevelopmental processes that had not been previously considered. Furthermore, by linking genetic findings to direct neurobiological markers of brain function using cognitive neuroscience and direct experimentation on model systems, the effects of genetic variation associated with ADHD on brain function can be better understood.

We therefore conclude that with the identification of known genes associated with ADHD, the goal of ADHD genetics is now shifting from gene discovery towards gene functionality, at the level of molecular mechanisms and brain processes, and a focus on interaction with environmental pathogens [58]. Quantitative genetic studies have led to a perceptual shift where ADHD is perceived as one or more quantitative traits that share genetic influences with other developmental, behavioural and cognitive traits; gene identification will shed further light on the mechanisms involved. In the study of psychiatric and behavioural disorders, molecular genetic studies have already confirmed a priori hypotheses for some disorders and identified novel genes for others, and identification of many additional behavioural genes is expected in the coming decade. Combining genetic, environmental and neurobiological research strategies has the potential to delineate the causal links between behavioural traits, psychiatric conditions and developmental course, including the persistence/desistance of psychiatric symptoms and co-morbidity between psychiatric disorders and traits. As a result, we can look forward to new insights and potential advances in our treatment of ADHD throughout the lifespan.

## Competing interests

The author(s) declare that they have no competing interests.

## Authors' contributions

This article is based, in part, on presentations by each of the authors at the 16^th ^Eunethydis meeting, Valencia, 6–9 October 2005. JK, BN and PA drafted the manuscript, with further contributions from WC and SF. All authors read and approved the final manuscript.
